# Mental Health in Injured Athletes: A Narrative and Conceptual Review of Risk-Stratified Psychological Monitoring After Musculoskeletal Injury and Concussion

**DOI:** 10.7759/cureus.110533

**Published:** 2026-06-09

**Authors:** Harmon Colvett, Brooke Reinagel, Jacob Deeter

**Affiliations:** 1 Medical School, University of Tennessee Health Science Center, Memphis, USA; 2 Kinesiology, University of Missouri, Columbia, USA; 3 College of Health Sciences, University of Missouri, Columbia, USA

**Keywords:** athletes, concussion, mental health, rehabilitation, repetitive head impacts, return to sport, sport injury

## Abstract

Athletic injury is a biopsychosocial event rather than a purely mechanical one. Athletes recovering from musculoskeletal injury or sport-related concussion may experience depressive symptoms, anxiety, sleep disruption, fear of reinjury, role disruption, and reduced psychological readiness for return to sport. This article is explicitly presented as a narrative and conceptual review, not as a systematic or scoping review. Its aims are threefold: to synthesize the literature on mental health outcomes after athletic injury; to identify patterns by injury type, age group, competition level, and career stage; and to introduce a conceptual prototype, the Athlete Mental Health Change Index (AMHCI). The reviewed evidence supports several cautious conclusions. First, musculoskeletal injury is consistently associated with short-term psychological distress and impaired return-to-sport confidence. Second, concussion is associated with mood and recovery-related psychological symptoms, while repetitive head-impact exposure and later-life psychiatric outcomes remain associated in some studies but are limited by heterogeneous methods, recall bias, and confounding. Third, psychological response is moderated by premorbid mental health, coping style, athletic identity, social support, and environmental pressure to return prematurely. The AMHCI is presented as a provisional, hypothesis-generating framework rather than a validated clinical instrument. Its five domains and weighting coefficients were derived from theory and an illustrative literature review, not from Delphi consensus, content validity testing, pilot testing, or psychometric calibration. Accordingly, the model should not be used for diagnostic or return-to-sport decision-making without future validation. A validation roadmap is proposed, including assessment of content validity, factor structure, reliability, convergent validity, predictive validity, and threshold calibration.

## Introduction and background

Sport-related injury can alter far more than tissue integrity or competitive availability. It can disrupt identity, mood, sleep, social connection, confidence, and perceived control over the future. In sports medicine settings, these psychological responses matter because they can shape rehabilitation adherence, pain experience, symptom reporting, return-to-sport readiness, and reinjury risk [1-3].

The mental health consequences of injury are not uniform. Musculoskeletal injuries often generate distress through pain, loss of function, social isolation, and interruption of sport participation, whereas sport-related concussion may also involve acute cognitive and affective symptoms linked to brain injury [4-6]. Repetitive head impacts create a separate, broader exposure construct that may or may not culminate in diagnosed concussion, yet they are often conflated with concussion history in the literature [7,8]. Clarifying these constructs is necessary before proposing any risk-stratified monitoring model.

Several assessment approaches already exist in sports medicine and rehabilitation. Some are generic symptom screens, some are injury-specific return-to-sport readiness tools, and some are concussion symptom inventories. These tools are useful, but they do not fully address the problem considered here: repeated within-athlete monitoring across psychological domains before and after injury while also acknowledging differences in head-impact exposure [6,9-13].

This article, therefore, sharpens the research gap in two ways. First, it distinguishes between psychological screening for current symptom severity and surveillance of change from an individual athlete's own baseline. Second, it argues that the interpretation of change may warrant added caution in athletes participating in sports with greater routine head-contact exposure or greater cumulative concussion burden. This article does not claim that exposure weighting is already validated. Rather, it proposes a conceptual model that can be tested prospectively and interpreted cautiously in light of existing guidance on athlete mental health, rehabilitation, and concussion [3,6,7,9-12].

Operational definitions are necessary because several related constructs are often blended together in the literature. Sport-related concussion refers to a clinically recognized concussion occurring during sport or exercise participation. Repetitive head impacts refer to repeated head impacts occurring over time, including impacts that do not meet clinical concussion criteria. Head-trauma exposure refers to a pragmatic estimate of the athlete's likely head-impact environment based on sport context; it is not a direct biomechanical measurement. Concussion history refers to documented or credibly reported prior diagnosed concussions. Neurobiological vulnerability refers to a hypothesized increased susceptibility to adverse mood, cognitive, or recovery outcomes after head-impact exposure. This term does not imply established causation or neurodegenerative disease in an individual athlete.

This article is intentionally framed as a comprehensive narrative and conceptual review with illustrative literature support. It is not a systematic review, scoping review, meta-analysis, or review protocol. The purpose of the review was to integrate injury psychology, concussion literature, rehabilitation science, and athlete mental health guidance to refine a conceptual monitoring framework and identify the evidence strengths and uncertainties most relevant to that framework.

Literature identification was conducted from January 2025 through March 31, 2026, using PubMed, Google Scholar, and targeted hand-searching of sports medicine and sport psychology journals. Searches emphasized the prior 15 years but allowed inclusion of older foundational theory papers and landmark studies where directly relevant. Representative search strings included: ("athlete" OR "sport*") AND ("injury" OR "musculoskeletal injury") AND ("depression" OR "anxiety" OR "mental health"); ("sport-related concussion" OR concussion) AND ("depression" OR anxiety OR "mental health" OR recovery); ("repetitive head impact*" OR "head contact") AND athlete AND (psychiatric OR depression OR anxiety); and ("return to sport" OR rehabilitation) AND ("psychological readiness" OR "fear of reinjury" OR coping).

Included sources were peer-reviewed empirical studies, systematic reviews, meta-analyses, and major consensus statements addressing athletes, sport-related injury, concussion, repetitive head impacts, rehabilitation, psychological readiness, or mental health outcomes. Priority was given to studies involving adolescent, collegiate, elite, or retired-athlete populations. Studies focused exclusively on non-athlete populations, unrelated psychiatric topics, or injuries outside the sports context were excluded unless they provided a mechanistic or conceptual background directly necessary to interpret athlete findings.

The search and selection process was used to identify representative and conceptually important literature rather than generate an exhaustive census of all eligible studies. An initial search and title/abstract screening were performed by one author, followed by full-text review and conceptual relevance assessment by the authors. Disagreements regarding study inclusion were resolved through discussion. No formal dual-independent screening workflow, protocol registration, PRISMA (Preferred Reporting Items for Systematic Reviews and Meta-Analyses) flow diagram, or structured risk-of-bias tool was used. For that reason, the review does not present pseudo-systematic counts or claim reproducible study capture in the manner expected of a formal evidence synthesis. The different approaches are presented in Table 1. 

**Table 1:** Comparison of existing assessment approaches and the gap addressed by the proposed framework PHQ-9: Patient Health Questionnaire-9, GAD-7: Generalized Anxiety Disorder-7, ACL-RSI: Anterior Cruciate Ligament–Return to Sport after Injury, SCAT: Sport Concussion Assessment Tool, SRC: sport-related concussion, AMHCI: Athlete Mental Health Change Index.

Approach	Primary use	Strengths	Limitations for injured-athlete monitoring	Relevance to the present model
Generic distress screens (e.g., PHQ-9, GAD-7)	Brief cross-sectional screening for current depression/anxiety symptoms	Brief, familiar, and easy to score	Do not capture athletic identity, fear of reinjury, support, or individualized change from baseline	Useful adjuncts, but incomplete as sole monitoring tools
Injury-specific return-to-sport readiness scales (e.g., ACL-RSI)	Assess confidence/readiness after a specific injury or procedure	Strong relevance to rehabilitation and clearance discussions	Often injury-specific; limited coverage of broader mental health symptoms and premorbid baseline	Inform readiness, but not multidomain mental health surveillance
Fear of reinjury / kinesiophobia measures (e.g., Tampa Scale of Kinesiophobia)	Identify avoidance, fear, and movement-related threat appraisal	Useful when fear is the dominant recovery barrier	Narrow domain focus; may miss mood, support, sleep, and neurocognitive symptoms	Best as domain-specific supplements
Concussion symptom inventories (e.g., SCAT symptom checklists, postconcussion symptom inventories)	Track symptom burden after sport-related concussion	Relevant to acute concussion management	Usually not designed for broader rehabilitation psychology or long-term baseline comparison across injury types	Helpful after SRC, but not a universal injury-monitoring framework
Proposed AMHCI	Baseline-anchored multidomain surveillance before and after injury, with provisional exposure-informed interpretation	Integrates mood, anxiety, identity, coping/support, and neurocognitive-sleep domains	Not validated; weighting scheme and thresholds are provisional	Conceptual prototype intended for future empirical testing

The final narrative synthesis grouped evidence by injury category, population, competition level, career stage, and outcome domain. Findings were interpreted cautiously as stronger, mixed, or preliminary depending on consistency across studies, study design, and major sources of bias. Particular attention was paid to distinctions among association, plausible mechanism, and causal inference.

## Review

Musculoskeletal injury and psychological outcomes

Across the reviewed literature, musculoskeletal injury is the most consistently supported source of short-term psychological burden in athletes. Injured athletes commonly report depressive symptoms, anxiety, frustration, emotional lability, and reduced confidence during rehabilitation [1,4,5]. The evidence is particularly strong for associations between injury and lower psychological readiness for return to sport, greater fear of reinjury, and poorer rehabilitation engagement when coping resources are limited [2,13-15].

The clearest pattern is not that every injured athlete develops a mental disorder, but that injury creates a period of heightened emotional vulnerability. This distinction matters. The literature supports associations with distress and symptom burden far more strongly than it supports deterministic claims of psychiatric disorder caused by injury alone [3,9].

Evidence is strongest in anterior cruciate ligament (ACL) reconstruction and other major musculoskeletal injury cohorts, where self-efficacy, optimism, motivation, fear of reinjury, and perceived support appear relevant to both rehabilitation adherence and return-to-sport confidence [13-15]. The literature is weaker when attempting to quantify exact prevalence across all sports because injury severity, timing, psychological measures, and follow-up windows vary substantially.

The psychological burden of musculoskeletal injury is partly explained by the structure of athletic participation itself. For many athletes, injury removes access to daily routines, peer connections, training identity, competition goals, and external feedback. These losses are not merely secondary inconveniences; they can become central mechanisms of distress. An athlete with strong sport identification may experience even temporary removal from sport as a threat to self-worth, status, future opportunity, or belonging. For this reason, the same physical injury can produce different psychological consequences depending on athletic identity, competitive context, available support, and timing within a season or career.

Return-to-sport readiness is not identical to physical clearance. Athletes may demonstrate sufficient strength, range of motion, or functional performance on testing while still reporting fear, hesitation, loss of confidence, or avoidance. These psychological barriers matter because they can influence movement patterns, adherence, effort, symptom reporting, and actual return-to-play behavior [2,13-15]. The literature therefore supports a rehabilitation model in which psychological readiness is assessed alongside physical recovery rather than treated as a separate or optional concern.

At the same time, psychological monitoring should not pathologize ordinary reactions. Sadness, frustration, worry, and impatience are expected after injury. The clinical question is not whether an athlete experiences distress, but whether that distress is severe, persistent, worsening, functionally impairing, or disproportionate relative to the expected rehabilitation course. A baseline-anchored model may help distinguish temporary emotional fluctuation from meaningful psychological change.

Concussion, repetitive head impacts, and mental health outcomes

The concussion literature supports a cautious association between sport-related concussion and acute or subacute mood symptoms, particularly when symptom burden is high or when premorbid mental health difficulties are present [6,16,17]. The evidence also suggests that subacute headaches, depressive symptoms, and prior mental health problems may accompany slower recovery trajectories [17,18].

Sport-related concussion differs from many musculoskeletal injuries because cognitive, affective, vestibular, sleep, headache, and exertional symptoms may arise as part of the injury presentation itself [6,16,17]. Mood symptoms after concussion may reflect multiple overlapping mechanisms, including direct neurobiological disruption, sleep disturbance, pain, removal from sport, anxiety about recovery, academic or occupational stress, and premorbid psychological vulnerability. Functional neuroimaging and mechanistic traumatic brain injury literature support the plausibility of altered cerebral function after mild and moderate traumatic brain injury, but such evidence does not translate into simple clinical predictions for individual athletes [17,19,20].

The Amsterdam consensus statement on concussion in sport emphasizes multimodal assessment and individualized management rather than single-factor decision-making [6]. This is important for the present framework. A psychological monitoring tool should not replace concussion evaluation, neurocognitive assessment, graded exertional progression, clinical examination, or return-to-sport protocols. Instead, psychological monitoring should be considered an additional layer of care, especially when symptoms persist, when mood symptoms are prominent, or when an athlete's recovery trajectory deviates from expectations.

By contrast, the literature on repetitive head impacts and later-life psychiatric outcomes is more heterogeneous. Several studies of retired athletes and former football players report associations between greater cumulative exposure and later-life depression, apathy, executive dysfunction, or cognitive symptoms [19-21]. However, these studies are often retrospective, male-dominated, and vulnerable to recall bias, selection bias, imprecise exposure measurement, and residual confounding. Broader traumatic brain injury meta-analytic findings and population-based concussion studies support concern but do not justify simple causal claims in athlete cohorts [8,22]. The current evidence base therefore supports concern and further study, but not the claim that repetitive head impacts inevitably produce later psychiatric disease.

This distinction should remain explicit throughout interpretation. Clinical concussion, subconcussive exposure, and broad participation in collision sports are overlapping but nonidentical constructs. They should not be used interchangeably. A football player with no diagnosed concussions may still have substantial repetitive head-impact exposure. A soccer player may have one diagnosed concussion but a relatively different exposure history depending on position, level, heading frequency, and duration of play. A swimmer may have low routine head-contact exposure but still sustain a concussion through an accidental fall or collision. These examples illustrate why exposure categories should be treated as provisional, pragmatic approximations rather than precise biomechanical classifications.

The AMHCI framework therefore treats sport exposure and concussion history as interpretive modifiers rather than diagnostic determinants. The point is not to assign certainty where the literature does not support certainty. Rather, the goal is to encourage more cautious interpretation when an athlete has a higher plausible head-impact burden, especially if psychological symptoms worsen meaningfully from baseline.

Patterns by age group, competition level, sex, and career stage

Adolescent data suggest that concussion history is associated with later depression diagnoses or symptom burden in some samples, but much of this evidence is cross-sectional and relies on self-report [18,23]. Adolescents may be particularly sensitive to injury-related disruption because sport participation often intersects with school identity, peer belonging, parental expectations, recruitment aspirations, and developmental vulnerability. At the same time, adolescents may have less autonomy regarding treatment access, disclosure, transportation, scheduling, or engagement with mental health care. These contextual features make adolescent injury recovery psychologically distinct from adult or professional sport settings.

Collegiate and elite athletes show consistent relevance of psychological readiness, fear of reinjury, coping, and perceived support during rehabilitation [3,13-15]. Collegiate athletes may experience a dual burden of athletic and academic pressure. Injury may threaten scholarship status, team role, professional aspirations, or self-concept while also interfering with coursework and social life. Elite athletes may face even stronger institutional incentives to return quickly, and the psychological meaning of injury may be amplified by public scrutiny, contract implications, selection pressure, or career stage.

Retired-athlete studies more often address long-term depression, anxiety, or neurobehavioral symptoms, especially in former collision-sport athletes with extensive exposure histories [11,19,21]. These studies are important but difficult to interpret. Retirement itself can involve identity transition, loss of structure, chronic pain, financial stress, reduced social support, and changing health behaviors. In retired-athlete cohorts, cumulative head-impact exposure may interact with many nonneurological variables. Therefore, associations between exposure and later-life symptoms should be interpreted as signals requiring careful prospective study rather than as established causal pathways.

Female athlete representation remains limited in many head-impact studies, especially retired contact-sport cohorts, making generalization difficult. Sex differences are sometimes observed in baseline symptoms or reporting patterns, but the literature is not yet strong enough to support uniform sex-specific conclusions across all injuries and sports [11,18]. This is a material limitation of any proposed monitoring framework. A tool intended for broad athlete use must eventually be validated across sex, age, sport, competition level, injury type, and cultural context.

Competition level also changes interpretation. Adolescents may be more dependent on family and school context, elite athletes may face greater identity foreclosure and institutional pressure to return, and retired athletes bring cumulative exposure and life-course confounders that differ from those in active rehabilitation settings [3,9,21]. These differences argue against a one-size-fits-all interpretation of postinjury psychological symptoms. The same raw score change may carry different practical meaning depending on baseline functioning, sport environment, injury severity, support structure, and recovery context.

Psychological determinants of rehabilitation and return to sport

The strongest practical theme in the rehabilitation literature is that recovery is shaped by more than tissue healing. Athletic identity can magnify distress when participation is interrupted [1,24]. Fear of reinjury can persist after physical clearance [2,13,15]. Maladaptive coping, rumination, social isolation, sleep disruption, and pressure from coaches or institutions can worsen adjustment, whereas perceived support, self-efficacy, adaptive coping, and transparent communication may support better recovery trajectories [3,14]. Mindfulness-oriented work in athlete populations also suggests that modifiable psychological processes deserve attention even when the primary clinical problem is physical injury or performance disruption [25].

These findings support routine psychological attention during rehabilitation, but they do not require overmedicalizing ordinary emotions. Temporary disappointment, frustration, and worry are common after injury. The clinical task is to identify when symptom magnitude, persistence, or functional impact suggests that an athlete is diverging from an expected recovery trajectory rather than simply experiencing a normal transient response [3,12].

A structured model may improve communication among athletes, athletic trainers, physicians, coaches, and mental health professionals. Without a baseline, clinicians may struggle to interpret whether a postinjury score represents a meaningful change or merely reflects the athlete's usual emotional profile. A highly anxious athlete may score high at both baseline and after injury, whereas a typically stable athlete may show a smaller absolute score that represents a much larger personal change. Baseline anchoring addresses this limitation by making the athlete their own comparator.

This is especially relevant for athletes who underreport symptoms. Athletes may minimize distress because they fear losing playing time, disappointing coaches, appearing weak, jeopardizing recruitment, or being labeled as unreliable. Social norms and team culture influence reporting behavior, including concussion reporting [26-28]. A repeated, normalized, confidential monitoring process may reduce stigma by making psychological check-ins part of standard injury care rather than a special intervention reserved for crises. A summary of selected studies is presented in Table 2. 

**Table 2:** Analytical summary of representative evidence discussed ACL: anterior cruciate ligament, NFL: National Football League.

Study	Design	Population/sample	Sex/level	Exposure/injury	Outcome	Follow-up	Main contribution	Major limitations
Chrisman & Richardson, 2014 [5]	Cross-sectional analysis	36,060 adolescents; school/community sample	49.2% female	History of concussion	Current depression diagnosis	Single time point	Concussion history associated with higher depression prevalence	Self-reported concussion history; cross-sectional design; not sport-exclusive
Covassin et al., 2012 [6]	Cross-sectional baseline study	1,616 athletes (837 collegiate; 779 high school)	Male and female athletes	Baseline concussion-related symptom context	Depression scores and neurocognitive performance	Single time point	Sex and age differences observed in baseline symptom profiles	Not a postinjury recovery study; baseline design limits causal inference
Everhart et al., 2015 [7]	Systematic review	Multiple ACL reconstruction studies	Predominantly young adult surgical cohorts	Major musculoskeletal injury	Rehabilitation adherence, return to sport, knee outcomes	Varied by included study	Psychological variables such as self-efficacy, optimism, and coping were clinically relevant	Heterogeneous studies and measures; limited pooled quantification
Fralick et al., 2016 [8]	Longitudinal cohort	235,110 adults with concussion in Ontario	About half men	Community concussion	Long-term suicide risk	Administrative follow-up over years	Included as contextual non-athlete evidence that concussion can carry longer-term psychiatric associations	Not athlete-specific; residual confounding possible; included here as contextual evidence rather than direct injured-athlete rehabilitation evidence
Guskiewicz et al., 2007 [12]	Cross-sectional survey	2,552 retired NFL players	All male retired professionals	Recurrent concussion history	Depression diagnosis	Retrospective lifetime exposure	Greater concussion history associated with higher depression risk	Recall bias; self-reported exposure; retired male football sample only
Iverson et al., 2017 [15]	Systematic review	101 studies	Mixed ages and sports	Sport-related concussion	Predictors of clinical recovery	Varied by included study	Greater acute/subacute symptoms and prior mental health problems predicted slower recovery	Substantial methodological heterogeneity
Montenigro et al., 2017 [21]	Cross-sectional cohort	93 former football players (17 high school; 76 college)	All male former players	Cumulative head-impact exposure	Depression, apathy, executive dysfunction, cognition	Later-life single assessment	Higher estimated exposure associated with worse later-life symptoms	Small sample; all male; retrospective exposure estimation
Gouttebarge et al., 2019 [11]	Systematic review and meta-analysis	Meta-analyses of 1,579 to 1,686 former elite athletes	Sex composition varied, often male-dominant	Current and former elite sport participation	Distress, anxiety/depression, sleep disturbance, alcohol misuse	Varied by included study	Mental health symptoms occurred in both current and former elite athletes	Heterogeneous populations and outcome measures

Conceptual framework: Athlete Mental Health Change Index (AMHCI)

The AMHCI is presented here as a conceptual prototype. It is not a validated diagnostic scale, not a substitute for clinical judgment, and not a stand-alone return-to-sport tool. The AMHCI is introduced for the first time in this study and has no prior published source; its domains were conceptually derived from sport injury-response theory, return-to-sport readiness literature, concussion recovery research, and athlete mental health guidance (Appendices) [3,13,17,26,29].

The proposed framework was developed by identifying recurring constructs across the reviewed literature and established conceptual models of sport-injury response, including affective distress, anxiety and arousal, identity disruption, coping and support, and neurocognitive or sleep-related symptoms [3,13,17]. These constructs were then organized into a brief surveillance format intended to be practical for preseason baseline assessment and postinjury reassessment.

No patient or public involvement, formal expert-panel review, Delphi process, content-validity study, cognitive interviewing study, pilot administration, or psychometric validation was conducted before the development of this framework. That absence is important. It means that the AMHCI should be interpreted as hypothesis-generating and provisional rather than as a clinical tool ready for implementation or a validated instrument.

Domain generation and item structure

The five domains were selected because they recur across sport-injury response theory, return-to-sport readiness work, concussion recovery literature, and athlete mental health consensus guidance [3,13,17,26]. There is a slight overlap in each domain, but they clearly focus on different aspects of mental health and provide a check on one another to assess internal consistency between responses. Each domain contains four items for pragmatic balance: enough breadth to avoid single-item overdependence, but brief enough for repeated administration in sport settings. The equal four-item structure was chosen for symmetry and feasibility, not because factor analysis has demonstrated that four items are optimal in every domain. These different domains are presented in Table 3.

**Table 3:** Proposed domains of the AMHCI AMHCI: Athlete Mental Health Change Index.

Domain	Illustrative content	Conceptual rationale	Draft scoring
Mood and affective stability	Sadness, anhedonia, emotional lability, hopelessness	Captures depressive symptom burden and affective disruption commonly reported after injury	4 items; score 0-16
Anxiety and cognitive arousal	Excessive worry, racing thoughts, tension, fear of reinjury	Targets threat appraisal and arousal that may affect rehabilitation and confidence	4 items; score 0-16
Identity and role vulnerability	Athletic identity dependence, role loss, pressure to return	Addresses disruption of self-concept and external performance pressure	4 items; score 0-16
Coping capacity and perceived support	Confidence in handling setbacks, social support, isolation, stress management	Captures modifiable contextual and resilience factors	4 items; score 0-16
Neurocognitive and sleep-related symptoms	Mental fog, slowed thinking, concentration difficulty, sleep/fatigue symptoms	Allows overlap with concussion-related and stress-related symptom clusters	4 items; score 0-16

The mood and affective stability domain is intended to capture the emotional symptoms most commonly described after injury, including sadness, loss of interest, emotional instability, and hopelessness about future athletic participation or performance. This domain does not presume major depressive disorder. Rather, it identifies affective change that may warrant further conversation or clinical evaluation if substantial, persistent, or functionally impairing.

The anxiety and cognitive arousal domain targets excessive worry, racing thoughts, physical tension, and fear of reinjury. This domain is especially relevant because fear and threat appraisal can influence rehabilitation adherence, willingness to load the injured area, movement confidence, and return-to-sport decision-making. Fear of reinjury may persist even after objective recovery milestones are met [2,13,15].

The identity and role vulnerability domain reflects the importance of athletic identity in injury response. Athletes who strongly define themselves through sport may experience injury as a disruption of self-concept, social role, and future trajectory. This domain also includes perceived pressure to return before readiness because external pressure can distort symptom reporting and complicate clinical decision-making [3,24,27].

The coping capacity and perceived support domain captures modifiable protective and risk factors. Confidence in handling setbacks, perceived support from coaches and teammates, family support, stress management, and social connection may buffer injury-related distress. Conversely, isolation and poor coping capacity may worsen emotional symptoms and reduce rehabilitation engagement [3,14].

The neurocognitive and sleep-related domain allows the model to include symptoms that may overlap across concussion, pain, stress, and recovery disruption. Concentration difficulty, slowed thinking, mental fog, sleep disturbance, and fatigue are not specific to concussion, but they are clinically important because they can influence school, work, rehabilitation, and daily functioning [6,16,17].

Scoring logic and interpretation

Each proposed item is scored from 0, meaning not at all, to 4, meaning severe. Domain scores range from 0 to 16, and the total raw score ranges from 0 to 80. Higher scores indicate greater symptom burden.

The framework is baseline-anchored. Each athlete completes the questionnaire before the season or before exposure-intensive participation begins. After injury, the athlete is reassessed at clinically appropriate intervals, such as the acute phase, mid-rehabilitation, and before return-to-sport clearance discussions.

The raw change score is defined as the postinjury total score minus the baseline total score. Under this definition, positive values indicate worsening relative to baseline, zero indicates no net change, and negative values indicate improvement. The same directionality applies to domain-specific change scores. This corrects the internal inconsistency in the prior draft, in which the sign of change and the interpretation thresholds did not align.

Worked example: if an athlete's baseline total is 12 and the postinjury total is 20, the raw change score is +8. If the athlete participates in a moderate-contact sport with a proposed exposure modifier of 1.15 and has one prior concussion with a proposed concussion-history modifier of 1.10, the adjusted change score is 8 × 1.15 × 1.10 = 10.12. Under the provisional thresholds proposed here, that pattern would indicate moderate worsening requiring structured psychological evaluation rather than routine monitoring alone.

The purpose of the adjusted score is not to diagnose a mental health condition. Instead, it is intended to support triage, communication, and research testing. The model assumes that a change from baseline may have different clinical significance depending on head-impact context and concussion history. However, the proposed coefficients are illustrative and must not be treated as validated risk weights.

Exposure categories and provisional modifiers

The sport-exposure classification is intentionally framed as a pragmatic preliminary categorization, not a precise biomechanical taxonomy. Head-impact exposure varies by position, sex, age, training load, competition level, protective equipment, and rule set. Thus, a single sport label cannot fully capture exposure. The categories below are offered only to structure future research and should be refined using published exposure data whenever the model is tested prospectively. The benefit of a modified structure such as the one presented here is that it allows counselors and other professionals to weigh different situations with an appropriate degree of professional judgment.

Suggested categories are low routine head-contact exposure, intermittent head-contact exposure, and high or frequent head-contact exposure. Examples of low routine head-contact exposure include swimming, track and field, distance running, and rowing. Examples of intermittent head-contact exposure include soccer, basketball, baseball or softball, and wrestling. Examples of high or frequent head-contact exposure include American football, rugby, ice hockey, boxing, and mixed martial arts. Providers and investigators should override sport-level classification when position, rule set, or participation pattern clearly changes exposure.

The proposed exposure multipliers of 1.00, 1.15, and 1.30 and concussion-history multipliers of 1.00, 1.10, 1.30, and 1.60 are best understood as literature-informed heuristic coefficients. They are not empirically calibrated weights. Their value in this study is conceptual: they operationalize the idea that interpretation of symptom worsening may warrant greater caution in athletes with greater plausible head-impact burden [6,7,19,20]. Future studies must determine whether these coefficients should be revised, attenuated, removed, or replaced entirely. Examples of different variables and their multipliers are presented in Table 4. 

**Table 4:** Provisional modifiers used in the conceptual model

Variable type	Subcategory	Multiplier	Notes
Sport exposure modifier	Low routine head-contact exposure	1.00	Illustrative examples: swimming, track and field, rowing
Sport exposure modifier	Intermittent head-contact exposure	1.15	Illustrative examples: soccer, basketball, baseball/softball, wrestling
Sport exposure modifier	High or frequent head-contact exposure	1.30	Illustrative examples: football, rugby, ice hockey, boxing, mixed martial arts
Concussion-history modifier	0 prior concussions	1.00	Reference category
Concussion-history modifier	1 prior concussion	1.10	Heuristic coefficient
Concussion-history modifier	2 prior concussions	1.30	Heuristic coefficient
Concussion-history modifier	3 or more prior concussions	1.60	Heuristic coefficient

These provisional threshold bands were selected as heuristic interpretation ranges for conceptual demonstration and have not been empirically calibrated against clinical outcomes. The adjusted score change and resultant actions are shown in Table 5.

**Table 5:** Provisional interpretation thresholds for adjusted worsening scores

Adjusted change score	Interpretation	Suggested action
≤0	No worsening or improvement relative to baseline	Continue standard clinical monitoring; interpret clinically
1-4	Minimal worsening	Routine monitoring and repeat reassessment if symptoms persist
5-9	Mild worsening	Increase observation; consider targeted supportive intervention
10-17	Moderate worsening	Structured psychological evaluation or referral discussion
≥18	Substantial worsening	Referral to a licensed mental health professional or urgent escalation as clinically indicated

Clinical meaning of baseline-anchored change

The central conceptual contribution of the AMHCI is not that it invents entirely new psychological constructs. Mood, anxiety, coping, social support, athletic identity, and sleep-related symptoms already appear across sports medicine and psychology literature [1-3,13,17,24,26]. The contribution is the proposed organization of these constructs into a repeated, baseline-anchored format that emphasizes change over time.

This distinction matters because cross-sectional symptom screens and change scores answer different clinical questions. A cross-sectional screen asks, "How severe are the athlete's symptoms right now?" A baseline-anchored change model asks, "How much has this athlete changed from their own usual state?" Both questions are useful, but they are not interchangeable.

For example, an athlete with mild baseline anxiety may show a small absolute increase that still reflects a meaningful personal change. It is important to acknowledge the possibility of a moving baseline. Repeated baseline testing, usually conducted at the start of each season, strives to assess the baseline as it changes over time. Moreover, it is important for a counselor to recognize a moving baseline in an effort to ensure that mental health problems are not developing quietly. Another athlete with high baseline distress may have a high postinjury score but little change from baseline. The first athlete may need early support despite a modest absolute score, while the second may need ongoing care, but not necessarily because of the injury alone. A monitoring framework that includes both absolute burden and within-athlete change could be more informative than either approach alone.

Baseline anchoring also helps reduce interpretive bias. Clinicians may otherwise rely on subjective impressions of personality, toughness, sport type, or expected emotional response. A structured monitoring process does not remove clinical judgment, but it gives teams a more consistent starting point for conversation.

Clinical implementation safeguards

Any real-world implementation of routine psychological monitoring requires safeguards. Confidentiality must be clearly defined before preseason screening begins. Athletes should understand who can see their responses, how the data will be stored, and under what circumstances information will be shared [3,27,29].

Referral pathways must also be predetermined. A monitoring tool has limited value if elevated scores do not trigger timely access to a licensed mental health professional, team physician, athletic trainer, school counselor, or emergency services when appropriate [3]. A score should never create responsibility without a response plan.

False positives and stigma must be anticipated. Not every emotional reaction to injury reflects psychopathology. Monitoring should be used to support conversation, triage, and trend recognition rather than to label athletes prematurely. Trust is particularly important in environments where athletes fear being viewed as weak, unreliable, or medically disqualified [3,12,27].

Role boundaries should be explicit. Coaches can support culture and referral, athletic trainers and team physicians can integrate screening into injury management, and mental health professionals should evaluate and treat clinically significant concerns. A score should inform communication, not replace clinical assessment.

Implementation would also require attention to workflow. If the AMHCI or a similar tool is administered too often, athletes may experience survey fatigue. If it is administered too rarely, important changes may be missed. Practical implementation might include preseason baseline testing, reassessment after a clinically significant injury, periodic reassessment during prolonged rehabilitation, and reassessment before return-to-sport discussions. The timing should be adapted to the sport, setting, injury type, and available resources.

Ethical implementation also requires avoiding coercive use. Athletes should not perceive the tool as a hidden clearance test or as a mechanism for punishment. The safest framing is supportive: psychological monitoring is part of comprehensive injury care because injury affects the whole athlete. Data use should be transparent, minimal, clinically justified, and confidential whenever possible.

Limitations of the evidence and proposed framework

This study has several limitations. First, it is a narrative and conceptual review rather than a formal systematic review. The literature search was transparent but not exhaustive, and no formal risk-of-bias appraisal was performed. The review should therefore be interpreted as an integrative synthesis rather than a reproducible evidence map.

Second, the literature itself is heterogeneous across sports, age groups, injury definitions, follow-up intervals, outcome measures, and comparison groups. That heterogeneity limits pooled interpretation and weakens broad prevalence claims. Studies of ACL reconstruction, concussion recovery, retired football players, adolescent school samples, elite athlete cohorts, and general traumatic brain injury populations cannot be treated as interchangeable.

Third, many studies rely on self-reported concussion history or retrospective exposure estimates, creating recall and misclassification bias. This limitation is particularly important for repetitive head-impact research. Athletes may not accurately remember prior concussions, may not have reported them at the time, or may have sustained impacts that were never clinically recognized. Similarly, sport participation does not equate to precise biomechanical exposure.

Fourth, female athletes remain underrepresented in important parts of the head-impact literature, particularly in retired-athlete cohorts. This limits generalizability and makes it inappropriate to assume that findings from male collision-sport cohorts apply uniformly to female athletes or to athletes in different sport environments.

Fifth, the AMHCI has not undergone content validation, pilot testing, reliability analysis, factor analysis, or threshold calibration. Its domains and coefficients therefore remain provisional. The item pool may require substantial revision after expert review, athlete feedback, psychometric testing, and subgroup analysis.

Sixth, there is a real risk of pathologizing normal emotional responses to injury if the framework is used rigidly rather than clinically. For that reason, the proposed model should be treated as a structured surveillance concept for research development, not as a validated clinical decision instrument.

Finally, the model does not currently include all potentially relevant variables. Pain intensity, injury severity, time loss, socioeconomic stress, prior trauma, sleep history, medication use, academic strain, financial pressure, team culture, and access to care may all influence psychological response. Future versions of the model may need to include contextual modules or separate interpretive guidance for different settings.

Future directions and validation roadmap

Because the AMHCI is conceptual rather than validated, the next step in the literature should focus on staged instrument development rather than immediate clinical deployment. Table 6 summarizes the staged validation roadmap needed before the AMHCI could be considered for clinical or research implementation.

**Table 6:** Validation roadmap for the AMHCI AMHCI: Athlete Mental Health Change Index.

Validation domain	Suggested study design	Primary question
Content validity	Expert panel review, Delphi consensus, cognitive interviewing with athletes and clinicians	Clarify item wording, relevance, and comprehensiveness
Feasibility and acceptability	Pilot preseason and postinjury administration	Completion time, missingness, acceptability, stigma concerns
Internal structure	Exploratory and confirmatory factor analysis	Whether the proposed five-domain structure is supported
Reliability	Internal consistency and test-retest stability at baseline	Score precision and short-term stability
Convergent/discriminant validity	Compare with established distress, readiness, and concussion symptom measures	Whether the tool behaves as theoretically expected
Predictive validity	Prospective cohort studies after injury	Delayed recovery, rehabilitation adherence, return-to-sport timing, reinjury, persistent symptoms
Threshold calibration	Receiver operating characteristic and clinically anchored cut-points	Whether provisional categories map onto meaningful clinical actions
Subgroup calibration	Stratified analyses by sex, age, sport, position, and competition level	Whether weighting and thresholds should differ across populations

The first stage should involve content validity. Experts in sports medicine, athletic training, rehabilitation psychology, psychiatry, neuropsychology, and athlete mental health should review the proposed domains and items. Athletes should also participate in cognitive interviewing to determine whether the items are understandable, acceptable, and relevant. Without athlete input, the tool risks using language that is clinically sensible but practically ineffective.

The second stage should evaluate feasibility. A tool that is theoretically strong but too long, stigmatizing, confusing, or difficult to administer will not be used consistently. Pilot studies should measure completion time, missing data, athlete comfort, staff burden, and responses to repeated administration.

The third stage should examine the internal structure. The proposed five-domain framework is theoretically derived. Factor analysis is needed to determine whether the domains actually behave as separate but related constructs. It is possible that some domains will merge, split, or require item replacement.

The fourth stage should assess reliability. Baseline test-retest stability is particularly important because the model depends on detecting change. If scores fluctuate substantially in the absence of injury or meaningful clinical change, the tool will have limited interpretive value.

The fifth stage should examine convergent and discriminant validity. AMHCI mood and anxiety domains should correlate with established distress measures, while identity- and readiness-related domains should relate to existing sport-specific psychological readiness measures. Neurocognitive and sleep-related items may overlap with concussion symptom inventories but should not be assumed to replace them.

The sixth stage should test predictive validity. Prospective studies should determine whether baseline scores, raw change scores, domain-specific changes, or adjusted change scores predict rehabilitation adherence, prolonged symptoms, delayed return to sport, reinjury, mental health referral, or athlete-reported recovery quality.

The seventh stage should calibrate thresholds. The proposed cut-points are heuristic. Empirical work should determine whether these thresholds meaningfully separate athletes who need routine monitoring, targeted support, structured evaluation, or urgent referral. Receiver operating characteristic analysis, clinical anchors, and patient-centered outcomes may all be useful.

The eighth stage should examine subgroup calibration. Sex, age, sport, position, competition level, injury type, and concussion history may all affect score interpretation. A model validated only in one population should not be generalized broadly without further testing.

Future validation and clinical implementation considerations

Because the AMHCI is conceptual rather than validated, its most appropriate use at this stage is as a research framework for prospective testing rather than as a clinical instrument. Future studies should begin with content validation through expert review, Delphi methods, and cognitive interviewing with athletes, clinicians, athletic trainers, and mental health professionals. Feasibility and acceptability studies should then evaluate completion time, missing data, stigma concerns, confidentiality concerns, athlete understanding, and implementation burden. Psychometric studies should test internal structure, reliability, test-retest stability, convergent validity, discriminant validity, and predictive validity. Prospective cohort studies should determine whether baseline scores, raw change scores, domain-specific changes, or adjusted change scores predict rehabilitation adherence, delayed recovery, persistent symptoms, return-to-sport timing, reinjury, or the need for mental health referral. Thresholds should be calibrated using clinically meaningful anchors rather than assumed cut-points.

Future validation must also include subgroup-specific analyses. Sex, age, sport, position, injury type, competition level, prior concussion history, and access to care may all influence psychological response and score interpretation. A framework developed primarily from male collision-sport or elite-athlete data should not be generalized uncritically to female athletes, adolescent athletes, recreational athletes, non-contact athletes, or athletes in under-resourced settings. Similarly, the psychological meaning of injury may differ for an adolescent trying to maintain peer belonging, a collegiate athlete concerned about scholarship status, an elite athlete under institutional pressure to return, and a retired athlete interpreting symptoms through the lens of cumulative exposure and career transition.

Clinical implementation, if future evidence supports it, would require safeguards. Psychological monitoring must be confidential, clearly explained, and linked to predetermined referral pathways. Athletes should know who can access their responses, how data are stored, and what types of responses trigger further support. Coaches, athletic trainers, physicians, and mental health professionals should have clearly defined roles. A monitoring score should support conversation and clinical judgment, not replace either. Used improperly, such a tool could stigmatize athletes, discourage honest disclosure, or be misused as a clearance mechanism. Used appropriately, it could normalize psychological care, identify athletes whose recovery is complicated by distress, and promote earlier referral when symptoms exceed expected adjustment.

## Conclusions

Athletic injury should be understood as a biopsychosocial event rather than a purely mechanical interruption of sport participation. The available literature supports the view that musculoskeletal injury and sport-related concussion can affect mood, anxiety, sleep, identity, perceived support, coping capacity, and confidence in returning to sport. These psychological responses may influence rehabilitation adherence, symptom reporting, clinical communication, return-to-sport readiness, and reinjury risk. At the same time, the evidence does not support deterministic claims that all injured athletes develop psychiatric disorders or that repetitive head impacts inevitably cause later psychiatric disease. A balanced approach should recognize clinically meaningful distress while avoiding the overpathologizing of expected emotional responses to injury.

The AMHCI is proposed as a conceptual, baseline-anchored framework for organizing multidomain psychological monitoring after athletic injury. Its intended contribution is the identification of a practical research model that emphasizes within-athlete change, cautious exposure-informed interpretation, and structured follow-up. Until formal validation is completed, the AMHCI should be regarded as a hypothesis-generating prototype that promotes further investigation in the field. Future work should determine whether its domains, scoring structure, modifiers, and thresholds are reliable, valid, acceptable, and useful across different sports, injuries, ages, sexes, and competition levels.

## Appendices

Draft AMHCI item pool

The draft items below are provided so the conceptual prototype can be reviewed directly. They remain provisional and have not undergone content-validation testing, cognitive interviewing, pilot administration, or psychometric evaluation.

**Table 7:** Proposed AMHCI AMHCI: Athlete Mental Health Change Index.

Domain code	AMHCI domain	Item no.	Draft item
A1	Mood and affective stability	1	I have felt persistently sad or low in mood.
A1	Mood and affective stability	2	I have experienced a loss of interest or pleasure in activities I normally enjoy.
A1	Mood and affective stability	3	I have felt emotionally unstable or prone to sudden mood changes.
A1	Mood and affective stability	4	I have felt hopeless about my athletic future or performance.
A2	Anxiety and cognitive arousal	1	I have felt excessive worry related to sport participation or performance.
A2	Anxiety and cognitive arousal	2	I have experienced racing thoughts or difficulty calming my mind.
A2	Anxiety and cognitive arousal	3	I have felt physically tense or unable to relax.
A2	Anxiety and cognitive arousal	4	I have feared reinjury or worsening of symptoms during activity.
A3	Identity and role vulnerability	1	My identity feels strongly dependent on being an athlete.
A3	Identity and role vulnerability	2	I have felt lost or uncertain when unable to participate in sport.
A3	Identity and role vulnerability	3	I have felt pressure to return to play before feeling ready.
A3	Identity and role vulnerability	4	Injury would significantly diminish my sense of self-worth.
A4	Coping capacity and perceived support	1	I do not feel confident in my ability to cope with injury or setbacks.
A4	Coping capacity and perceived support	2	I do not feel supported by coaches, teammates, or family.
A4	Coping capacity and perceived support	3	I am not able to manage stress effectively.
A4	Coping capacity and perceived support	4	I have felt isolated when dealing with sport-related challenges.
A5	Neurocognitive and sleep-related symptoms	1	I have had difficulty concentrating or maintaining attention.
A5	Neurocognitive and sleep-related symptoms	2	I have experienced slowed thinking or mental fog.
A5	Neurocognitive and sleep-related symptoms	3	I have had difficulty falling or staying asleep.
A5	Neurocognitive and sleep-related symptoms	4	I have felt unusually fatigued during daily activities.
